# Stress decreases spermatozoa quality and induces molecular alterations in zebrafish progeny

**DOI:** 10.1186/s12915-023-01570-w

**Published:** 2023-04-03

**Authors:** David G. Valcarce, Marta F. Riesco, Leyre Cuesta-Martín, Anna Esteve-Codina, Juan Manuel Martínez-Vázquez, Vanesa Robles

**Affiliations:** 1 Cell Biology Area, Molecular Biology Department, Universidad de León, Campus de Vegazana s/n, 24071 León, Spain; 2 Instituto Español de Oceanografía, Centro Oceanográfico de Santander (COST-IEO), CSIC, Calle Severiano Ballesteros 16. 39004, Santander, Spain; 3 CNAG-CRG, Centre for Genomic Regulation, Barcelona Institute of Science and Technology (BIST), Barcelona, Spain; 4 Universitat Pompeu Fabra (UPF), Barcelona, Spain

**Keywords:** Zebrafish, Chronic stress, Testis, NMD, Sperm, Progeny

## Abstract

**Background:**

Chronic stress can produce a severe negative impact on health not only in the exposed individuals but also in their offspring. Indeed, chronic stress may be contributing to the current worldwide scenario of increasing infertility and decreasing gamete quality in human populations. Here, we evaluate the effect of chronic stress on behavior and male reproductive parameters in zebrafish. Our goal is to provide information on the impact that chronic stress has at molecular, histological, and physiological level in a vertebrate model species.

**Results:**

We evaluated the effects of a 21-day chronic stress protocol covering around three full waves of spermatogenesis in *Danio rerio* adult males. The induction of chronic stress produced anxiety-like behavior in stressed males as assessed by a novel tank test. At a molecular level, the induction of chronic stress consistently resulted in the overexpression of two genes related to endoplasmic reticulum (ER) stress in the brain. Gene set enrichment analysis (GSEA) of testes suggested a dysregulation of the nonsense-mediated decay (NMD) pathway, which was also confirmed on qPCR analysis. Histological analysis of the testicle did not show significant differences in terms of the relative proportions of each germ-cell type; however, the quality of sperm from stressed males was compromised in terms of motility. RNA-seq analysis in stress-derived larval progenies revealed molecular alterations, including those predicted to affect translation initiation, DNA repair, cell cycle control, and response to stress.

**Conclusions:**

Induction of chronic stress during a few cycles of spermatogenesis in the vertebrate zebrafish model affects behavior, gonadal gene expression, final gamete quality, and progeny. The NMD surveillance pathway (a key cellular mechanism that regulates the stability of both normal and mutant transcripts) is severely affected in the testes by chronic stress and therefore the control and regulation of RNAs during spermatogenesis may be affected altering the molecular status in the progeny.

**Graphical Abstract:**

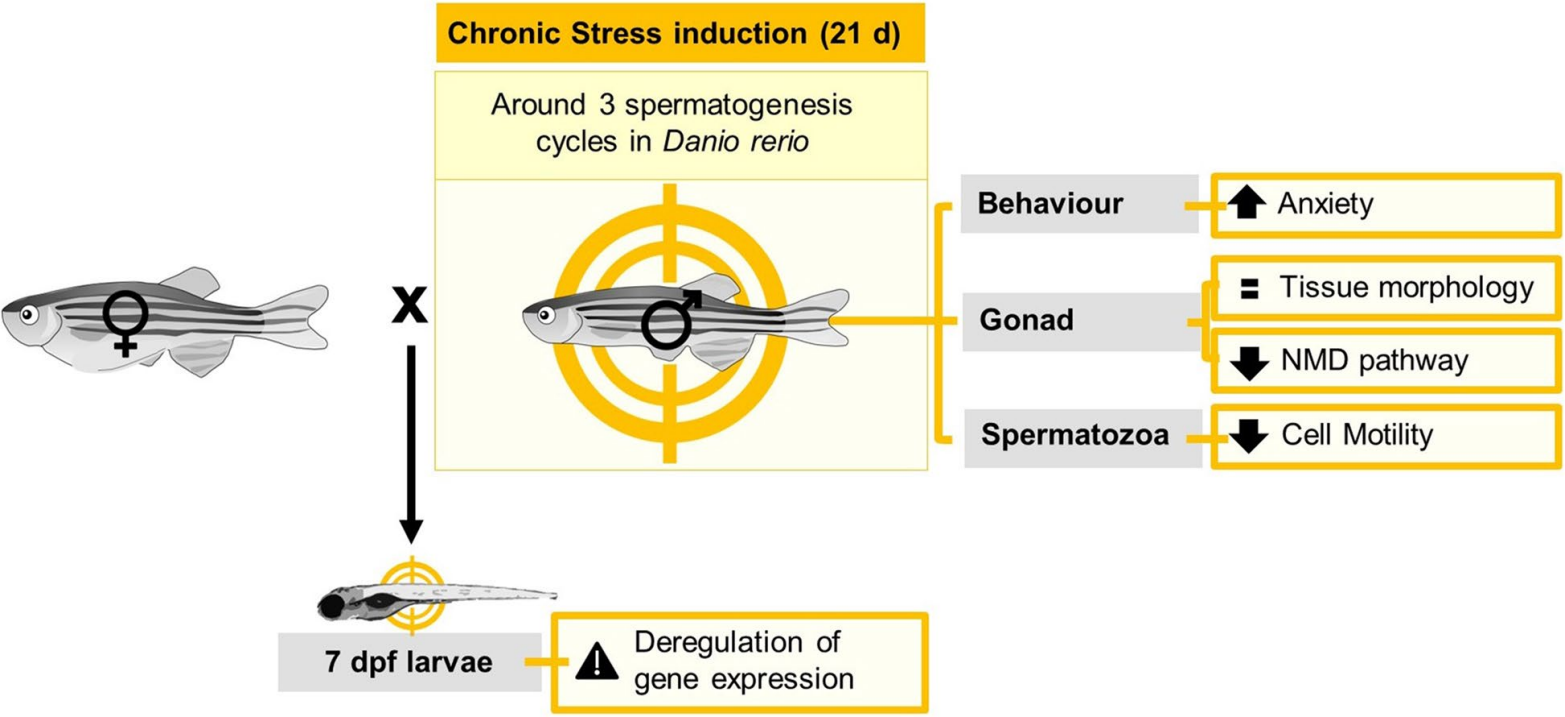

**Supplementary Information:**

The online version contains supplementary material available at 10.1186/s12915-023-01570-w.

## Background

In recent decades, a decline in human fertility has generated growing concern. Infertility is defined as the inability to become pregnant after 12 months of regular unprotected intercourse [1]. Nowadays, it is estimated that around 15% of couples suffer from infertility [2]. Male-factor infertility plays a role in 50% of involuntarily childless couples—single cause in 20% of cases and contributing factor together with female infertility in 30% [3, 4]. The increase in the prevalence of male infertility and poor semen quality has become a pressing public health issue, generating a huge volume of literature on its potential links with a variety of factors—lifestyle, genetic, or developmental—as male infertility represents a multifactorial pathological condition with highly heterogeneous phenotypic presentations.

Age, smoking, and alcohol consumption have been found to be significant risk factors for semen quality in both fertile and infertile men, but psychological stress can also represent an important factor to consider [5–7]. Stress is defined as a real or perceived threat from internal or external adverse events—or stressors—to the homeostasis or well-being of an organism [8, 9]. The potential relation of stress with infertility has been debated for years [10]. Stress can be acute—an organism response to adapt to a changing environment—or chronic—a recurrent stimulation of the stress response within a short timeframe leads to a variety of adverse health effects [11]. Chronic stress can have an important impact on health not only of the exposed individuals but also in their progeny. The intergenerational transmission of stress has been described as an effect in offspring as a result of parental stress exposures [12], and it has been reported to happen via gestational uterine environment [13] but also via gametes [14–16]. However, most of these studies were focused on the effects of maternal stress exposure on offspring, in particular during pregnancy [12].

The link between the stress system and the testicles has been previously described [17]; the neuroendocrine link between the hypothalamic–pituitary–adrenal (HPA) axis and the hypothalamic–pituitary–gonadal (HPG) axis can be easily affected by psychological stress [6]. Furthermore, if the cascade triggered by stress produces a dysregulation of the spermatogenesis process, this can also produce a decrease in sperm quality parameters (such as sperm motility and concentration) or produce morphological alterations [5].

Spermatogenesis involves its own interactions between somatic and germinal testicular cells, resulting in the self-renewal of spermatogonial stem cells (SSCs) and in their differentiation into highly specialized terminally differentiated spermatozoa. Spermatogenesis is a complex succession of molecular events, involving thousands of genes [18] under multiple levels of regulation. Therefore, a myriad of potential molecular pathways may be affected by stressors. The contribution of the spermatozoa and its RNAs in progeny transmission has been a topic of active debate [19]. There are some outstanding questions that have contributed to this skepticism on paternal contribution. Firstly, in terms of quantity, the size of spermatozoa and the mRNA payload comparing to the oocyte shed doubts on the importance of these molecules of paternal origin beyond fertilization [20]. Evidence of their importance has been provided in several studies that demonstrated that sperm cells can be a vehicle for certain phenotype transmission to the progeny [21, 22] and sperm-borne RNA contents are nowadays widely studied [23].

We hypothesize that chronic stress directly impacts key pathways in the vertebrate testis at molecular level, affecting the quality of the resulting gamete and progeny outcome. Our main objective is to provide more information on the negative impact that exposure to chronic stress has on male gonads. We intend to correlate changes in behavior derived from stress with alterations at molecular level in the testicle that can affect the quality of the final gamete and, therefore, the progeny.

To test our hypothesis, we use zebrafish (*Danio rerio*) as a model species. *D. rerio* is a favored vertebrate organism in reproduction science because this shares a close reproductive system with mammals in terms of regulation of the reproductive system [24, 25]. Due to the similarities between regulatory axes in zebrafish and human, the reproductive health status of the model under stress is highly predictive of mammalian responses [25] despite evolutionary distance and different reproductive strategies and spermatogenesis modes. In the present work, we exposed adult male zebrafish to chronic stress induction protocol. We measured fish behavior, brain gene expression, testis histology, transcriptomics, sperm motility, and resulting F1 progeny gene expression. The combination of behavioral, molecular, and histological studies provides a general scenario relevant for a better understanding of the effects of physiological stress on germ cells and reproduction.

## Methods

### Animal models

All experiments were performed in adult (8 months old) zebrafish (*Danio rerio;* AB wildtype strain) male siblings (weight: 0.3440 ± 0.0105 (g ± SEM)). Fish were raised and kept under standard laboratory conditions [26]. The batch of animals used in each trial are codified by a number for easier interpretation of the experimental design (batch 1; batch 2, batch 3, and batch 4). All males (within a batch) came from the same family.

### Experimental design

**Fig. 1:**
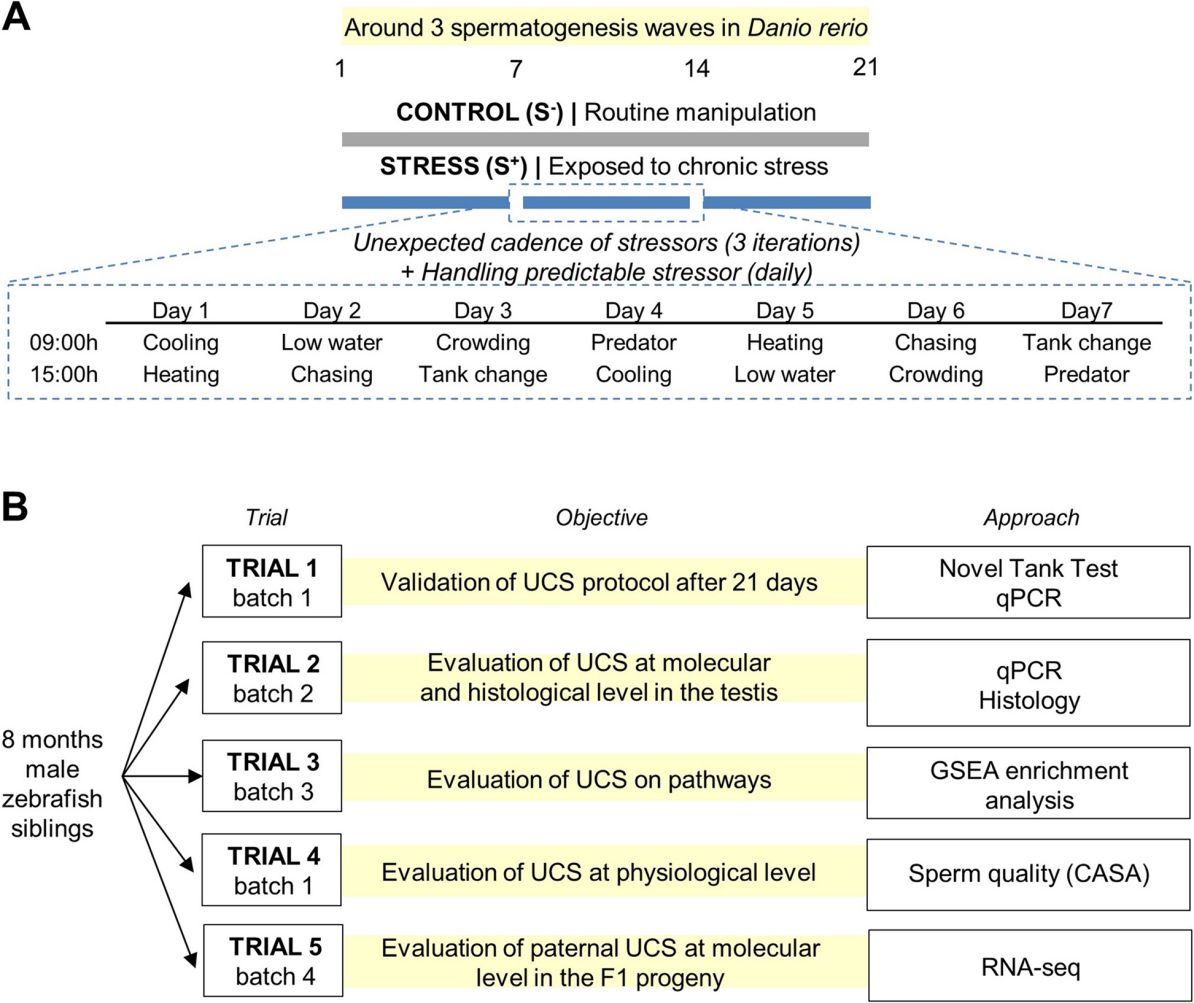
Experimental design. **A** Chronic stress (CS) protocol lasting 21 days to cover around three waves of spermatogenesis period in zebrafish. CS consisted of a predictable handling source of stress (twice per day) and a main body of unpredictable chronic stress (UCS). Each stressor included in the UCS was applied twice per week. Seven stressors were used in the UCS protocol: (1) cooling: 30 min in a tank at 23 °C; (2) heating: 30 min in a tank at 33 °C; (3) low water level: 2 min in a tank with extreme low water level exposing the dorsal body of the fish to the air; (4) chasing: 8 min of chasing with a net; (5) crowding: 50 min of crowding animals in a 250-mL beaker; (6) tank change: three consecutive relocations of the animals in a new tank after 30 min, and (7) predator: 50 min exposure to a video of the zebrafish predator *Archocentrus nigrofasciatus.* **B** Five trials performed to validate CS protocol (trial 1), perform molecular and histological analyses after CS protocol (trial 2), find altered pathways in the gonads after CS (trial 3), evaluate CS protocol at physiological level (trial 4), and analyze the molecular scenario in the derived progenies (trial 5). The fish batch used in each trial is codified by a number for easier interpretation of the experimental design (batch 1; batch 2, batch 3, and batch 4). CASA, computer-assisted sperm analysis

To generate contrasting physiological conditions in terms of stress, two culture conditions (control: S^−^; stress: S^+^) were used throughout 21 days (Fig. 1A), time of around three full rounds of spermatogenesis in zebrafish [27].

All batches were subject to the same chronic stress (CS) protocol. This protocol is based on a 7-day unpredictable chronic stress (UCS) protocol [28] with slight modifications. Briefly, animals in the S^+^ group were exposed to two stressors per day, in the morning and in the afternoon (Fig. 1). Each stressor was applied twice per week. Seven stressors were used in the UCS protocol: (1) cooling: 30 min in a tank at 23 °C; (2) heating: 30 min in a tank at 33 °C; (3) low water level: 2 min in a tank with extreme low water level exposing the dorsal body of the fish to the air; (4) chasing: 8 min of chasing with a net; (5) crowding: 50 min of crowding animals in a 250-mL beaker; (6) tank change: three consecutive relocations of the animals in a new tank after 30 min, and (7) predator: 50 min exposure to a zebrafish predator *Archocentrus nigrofasciatus*. In the present work, we replaced the real exposure to a specimen of *A. nigrofasciatus* with a video of this species projected on one of the walls of the induction tank (the effectiveness of this approach on zebrafish behavior is shown in Additional file 1: Fig. SM1). Stressors were applied in an induction tank. Therefore, this procedure should be considered as a potentially predictable stressor derived from handling. Then, the induced chronic stress in the S^+^ group should be understood as the sum of a main batch of unpredictable stressors and this handling predictable source of stress. In other words, the CS consisted of UCS plus the potentially predictable handling routine. The weekly sequence of stressors was repeated three times to cover the time frame of the design (21 days). Simultaneously, control fish (S^−^) were maintained undisturbed during the trials.

In a first trial (Fig. 1B), we used 18 male siblings (batch 1; 9 per experimental condition) for behavioral analysis to corroborate the efficiency of the induction of the chosen chronic stress (CS) protocol in the experimental setting. Brains from five males in each group were dissected for molecular analysis.

In a second trial (Fig. 1B), we created two new groups (batch 2; eleven males per group) and replicated the CS protocol as performed in our prior trial. One day after the end of the induction protocol, we euthanized S^−^ and S^+^ fish with a lethal dose (400 mg/L) of MS-222 and extracted their testes. From each group, six testicular samples were used for quantitative polymerase chain reaction (qPCR) gene expression analysis and the remaining five were fixed for histology analyses.

In a third trial (Fig. 1B), another 24 siblings (batch 3; 12 males per group) were exposed to the chronic stress protocol. Testes from these males were extracted and pooled for RNA sequencing (RNA-seq) analysis (see RNA extraction for RNA-seq analysis) and GSEA-based pathway enrichment analysis.

The fourth trial (Fig. 1B) was performed with the same individuals used in the behavioral analysis in Trial 1 (batch 1). Fifteen days before the beginning of the CS protocol, males were tagged with visible implant elastomers (VIEs) to track each individual before and after the trial. Red and green elastomers were prepared and visualized following the manufacturer’s recommendations (Northwest Marine Technology). We assigned an individual code number to each specimen according to the site of the VIE injection and color of the elastomer, as performed by our research group in a previous study [29]. To obtain an accurate representation of the impact of stress on sperm quality after approximately three cycles of spermatogenesis, we anesthetized all fish and extracted their semen for a first sampling 1 day (day −1) before the beginning of the CS protocol and, for a second sampling, again after 21 days of CS protocol. We arranged the experimental groups in two trials (Trials 1 and 4) according to total motility at day −1.

In the fifth trial, a new batch of 30 males (15 fish/group) was used (batch 4; Fig. 1B). The day after the ending of CS the protocol, both S^+^ and S^−^ males were crossed with control females (1♂:1♀ ratio). Spawning was induced in the morning as the light cycle was turned on. Fertilized embryos were rinsed and then randomly distributed in Petri dishes. The progeny resulting from each exposed male was considered as a biological replicate (*n* = 4–5). F1 animals were incubated at 28 ± 1 °C until 7 dpf, when larvae were sampled for RNA-seq.

### Effect of chronic stress induction protocol during three spermatogenic cycles on fish behavior

**Fig. 2:**
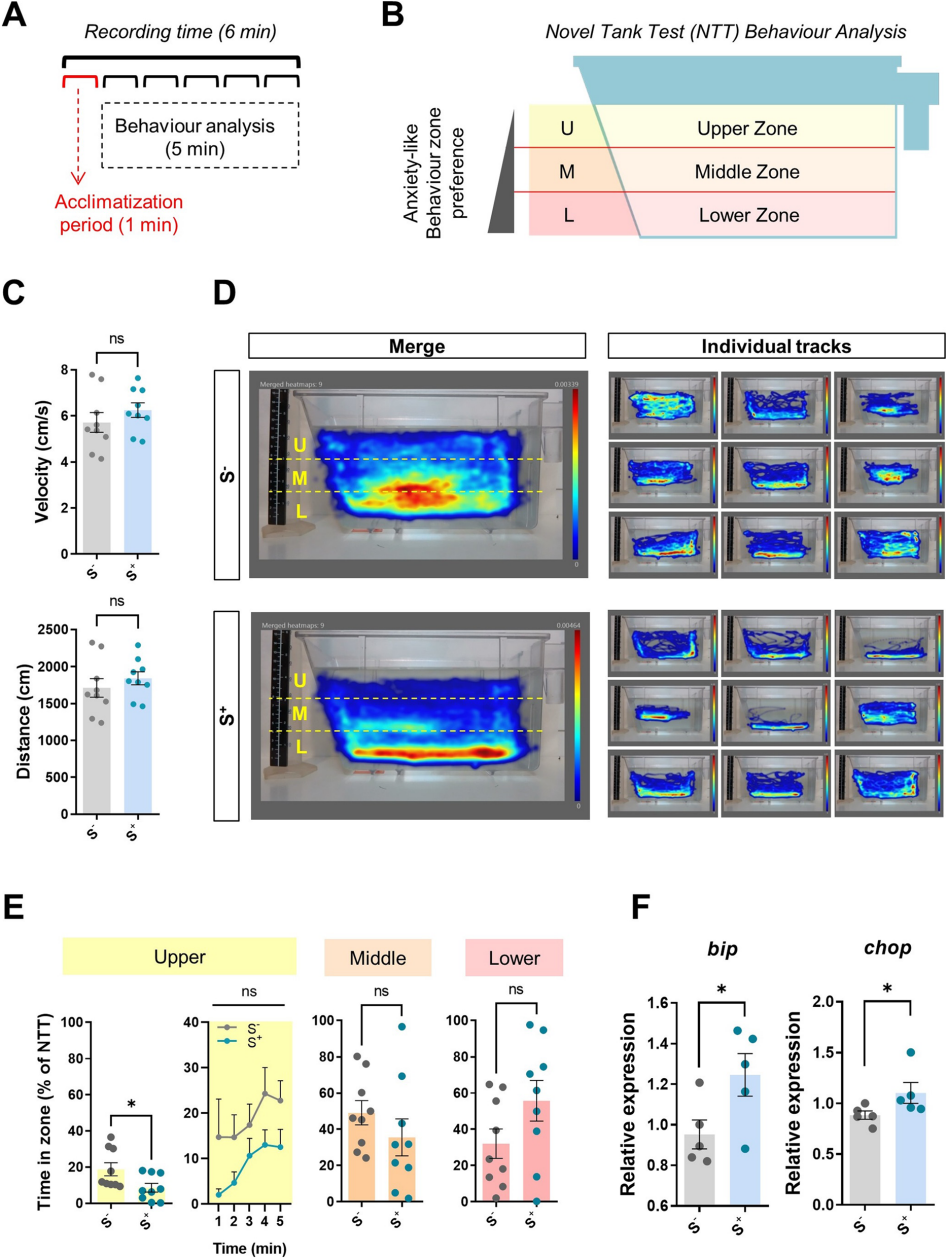
CS protocol validation. **A** Diagram of NTT experimental design (1-min acclimatization + 5-min evaluation). **B** Virtual zones (upper, middle, and lower) of evaluation area stablished with Noldus Ethovision® XT16 software*.* **C** Kinetic parameters: velocity (cm/s) and distance (cm). **D** Merge (*n* = 9) and individual heatmaps from both experimental groups showing the minimum amount of time an individual spent in each pixel in dark blue and the maximum in red. **E** Time spent by males in the upper, middle, and lower zones (%). **F** Relative gene expression in zebrafish brain of two genes involved in endoplasmic reticulum (ER) stress: *bip* and *chop*. S^−^: control males. S^+^: males exposed to the chronic stress (CS) protocol. Data are presented as mean ± SEM (kinetics and preference data: *n* = 9; gene expression analysis: *n* = 5; fish batch 1). *p < 0.0500

To evaluate the effects of the CS protocol on zebrafish anxiety levels, we performed a novel tank test (NTT) in trial 1 (Fig. 1B) after 21 days of exposure to stressors. On day 22, 1 day after the end of the CS protocol, fish were individually placed at the bottom of a trapezoidal transparent tank (width: 11 cm; height: 17.5 cm; length at top: 28 cm) containing 3.5 L of aquarium water. We changed the water after three trials to diminish variations in temperature (~28 °C) and to avoid presence of stress hormones from already trialed individuals. Zebrafish behavioral activity was recorded (1920 × 1080 px) for 6 min. The resulting videos were processed with Noldus Ethovision® XT16 (Noldus Information Technologies, Inc.) tracking software. The first minute was considered an acclimatization period and was not included in the analysis (Fig. 2A). The area was split into three virtual zones (upper, middle, and lower) to deliver an exhaustive evaluation of vertical swimming activity (Fig. 2B). Velocity of fish, distance swum, and time spent in the upper zone (percentage of NTT) were the endpoints analyzed. Heatmaps were generated to compare swimming patterns among group.

### Histology

In trial 2, after concluding CS protocol, testes were dissected, washed with phosphate-buffered saline (PBS), and fixed in 4% (w/v) PBS-buffered paraformaldehyde (4 °C, o/n). After a 30-min rinse with PBS, testis tissue was dehydrated and embedded in paraffin. Gonads were cut at serial sections of 5 μm, stained with hematoxylin eosin (H&E), and mounted with Entellan®. The sections obtained were morphometrically studied (magnification: × 400) under a light microscope (BX61, Olympus) and analyzed using image software (ImageJ). Images from nine nonoverlapping fields for each sample (five samples per treatment) were processed with a 540-point grid, as previously performed [30], to quantify the percentage of area occupied by the different germ cell types, based on the number of points counted over those germ cell types identified following morphological standards reported previously [27].

### RNA extraction

Two RNA extraction methods were used. Method 1 was used for batch 1 brains and batch 2 testes (for qPCR), while method 2 was used for batch 3 testes (for RNA-seq) and batch 4 larvae (for RNA-seq and qPCR validation).

#### Method 1

Total RNA was extracted using TRIzol Reagent (Invitrogen) according to the manufacturer’s specifications. Extractions were performed after NNT experiments in trial 1 and after the 21-day CS protocol was completed in trial 2. Lysis was performed using plastic pestles for mechanic disruption of tissues. Quality and quantity of RNA samples (A260/A280 ratio ranging from 1.8 to 2.0) were examined using a NanoDrop™ One/OneC spectrophotometer (ThermoScientific™). RNA integrity was evaluated using GelRed® to stain 28S and 18S ribosomal RNA (rRNA) fragments on 1% agarose in tris-acetate-EDTA (TAE) buffer (data not shown). Total RNA (1 μg) was reverse transcribed to complementary DNA (cDNA) using the High-Capacity cDNA Reverse Transcription Kit (Applied Biosystems) following the manufacturer’s protocol. The resulting cDNA samples were kept at − 20 °C prior to qPCR analysis.

#### Method 2

In trial 3, testes were pooled (three males per pool; batch 3) after the CS protocol, and in trial 4, 7dpf larvae from 4 different crossings per treatment were pooled (50 larvae per pool). Samples were processed using a first step of dissociation with Qiazol; a miRNeasy tissue kit (Qiagen) was then employed for RNA isolation. Quality of samples was examined using Experion (Experion™ Automated Electrophoresis System, BioRad) and a NanoDrop™ One/OneC spectrophotometer (ThermoScientific™). Only samples meeting the requirements (3 μg of RNA; RNA integrity number (RIN) > 8) were used in RNA-seq analysis. A testis replicate in the S^+^ group did not reach the requirements. In the 7 dpf larvae samples, for qPCR validation the reverse transcription was performed as previously described.

### RNA-Seq library preparation and sequencing

Stranded mRNA library preparation and sequencing were performed in the CNAG-CRG platform. The quantity and quality of the total RNA sample determined by Qubit RNA BR Assay kit (Thermo Fisher Scientific) and RNA 6000 Nano Bioanalyzer 2100 Assay (Agilent). The RNA-Seq libraries were prepared with KAPA RNA HyperPrep Kit with RiboErase (Roche) following the manufacturer´s recommendations using Illumina platform compatible adaptors with unique dual indexes and unique molecular identifiers (Integrated DNA Technologies). The final library was validated on an Agilent 2100 Bioanalyzer with the DNA 7500 assay. The libraries were sequenced on NovaSeq 6000 (Illumina) with a read length of 2 × 51 bp following the manufacturer’s protocol for dual indexing. Image analysis, base calling, and quality scoring of the run were processed using the manufacturer’s software Real Time Analysis (RTA v3.4.4) and followed by generation of FASTQ sequence files.

### RNA-Seq data processing and data analysis

RNA-seq reads were mapped against the *Danio rerio* reference genome (ENSEMBL release 104) using STAR version 2.7.8a [31] with ENCODE parameters. Genes were quantified with RSEM version 1.3.0 [32] with default parameters using the ENSEMBL release 104 annotation file. Differential expression analysis was performed with limma-voom [33] removing the unknown unwanted variation with sva [34]. Genes with an adjusted *p*-value < 0.0500 and |FC| > 1.5 were considered significant. Gene Set Enrichment Analysis (GSEA) was performed with a pre-ranked gene list based on moderated t statistic reported by limma-voom with the GSEA Preranked module [35, 36] and the REACTOME MsigDB. To facilitate data exploration, differentially expressed genes (DEGs) with a |FC| > 3 from larval progeny RNA-seq were used for g:Profiler [37] analysis (Gene Ontology (GO) biological process), and the resulting enriched biological processes were afterwards explored with g:Profiler for human phenotype ontology. R scripts used in this work can be found in Additional file 2: Script 1 and Additional file 3: Script 2.

### Quantification of messenger RNA (mRNA) expression levels by qPCR in brain, testis and larvae

We performed qPCR using SYBR green PCR master mix (Applied Biosystems) with a StepOnePlus (Applied Biosystems) thermocycler to quantify F0 mRNA expression levels of genes related to endoplasmic reticulum (ER) stress [38]—binding immunoglobulin protein (*bip*) and CCAAT/enhancer-binding protein homologous protein (*chop*)—seven genes involved in the nonsense-mediated mRNA decay (NMD) pathway—UPF1 RNA helicase and ATPase (*upf1*); UPF2, UPF3A, and UPF3B regulators of NMD (*upf2*,* upf3a*,* upf3b*); Tudor domain-containing protein (*tdrd6*); eukaryotic initiation factor 4E (*eif4a3*); and RNA-binding motif protein 8A (*rmb8a*)—and 4 NMD endogenous NMD substrates [39]: activating transcription factor 3 (*atf3)*, growth arrest and DNA-damage-inducible, beta (*gadd45b*), N-acetyltransferase 9 (GCN5-related, putative) (*nat9*), and transducin beta like 2 (*tbl2*). In the F1 larvae samples, 7 genes were selected based on RNAseq results for qPCR validation. These selected genes are as follows: N-myc downstream regulated 1b (*ndrg1b)*, centromere protein F3 *(cenpf3)*, calpain 3b *(capn3b)*, calymmin *(cmn)*, secretory calcium-binding phosphoprotein 9 *(scpp9)*, and solute carrier family 41 member 2a (*slc41a2a*). Primer pairs for NMD genes were designed against available mRNA sequences from the National Center for Biotechnology Information (NCBI) using Primer BLAST (www.ncbi.nlm.nih.gov/tools/primer-blast/). GenBank® references, primer sequences, amplicon length (bp), melting temperature (*T*m; °C), and efficiencies (%) are presented in Additional file 4: Table 1. Primers for *bip* and *chop* sequences were already published [40]. All replicates (brain: *n* = 5; testes: *n* = 5–6; progenies: *n* = 4) were performed in triplicate on 25 ng of cDNA in a 20-μL reaction consisting of 10 μL SYBR green master mix (Applied Biosystems) with forward and reverse primers (500 nM) and bidistillated water up to 20 μL. Thermal conditions were 10 min at 95 °C followed by 40 cycles of 10 s at 95 °C and 60 s at annealing temperature (60 °C). We performed a melting curve analysis to determine the specificity of qPCR reactions—one cycle at 95 °C for 15 s, 60 °C for 1 min, slow ramping of temperature to 95 °C, and 95 °C for 15 s. A no template control (NTC) was included in each run to guarantee absence of contamination. Gene expression was calculated relative to the housekeeping gene (HKG) *rps18* for testicular samples and *actb2* for brain and larvae samples (with the lowest coefficients of variation: 2.73 and 4.80 respectively provided by BestKeeper software; primers for HKGs were previously described [41, 42]), following Pfaffl's mathematical model [43]. Standard curves for target and reference genes were generated. Linearity, detection range, and qPCR amplification efficiency of each primer were analyzed prior to proceeding with samples (Additional file 4: Table 1).

### Zebrafish sperm collection and sperm quality evaluation

For trial 4, we used the same batch (batch 1) of 18 siblings used in the NTT experiment. Fish were visible implant elastomer (VIE) tagged 15 days before the start date of the CS protocol. Their sperm was collected one day before the start date of the CS protocol (day − 1). Fish were anesthetized by immersion in 168 mg/mL tricaine methanesulfonate (MS-222) solution dissolved in system water prior to sampling. The urogenital pore was dried, and sperm was collected with a pipette by bilateral abdominal pressure using fine forceps. Samples were collected avoiding urine contamination. The sperm obtained was 1:10 diluted in a 300-mosmol/kg Hank’s balanced salt solution (HBSS) nonactivating medium. The samples were kept at room temperature until computer assisted sperm analysis (CASA) evaluation. Males were relocated in individual fresh-water tanks to recover immediately after stripping and until motility data were processed.

Cell motility and concentration analyses were performed activating 1 μL of sperm with 9 μL of system water (28 °C) in a Makler chamber. Sample evaluation was performed using a phase-contrast microscope (Eclipse Ts2R, Nikon) with a × 10 negative contrast objective and a Basler A312fc digital camera (Basler Vision Technologies) set for 50 fps. Our endpoints—total motility, progressive motility (percentage of spermatozoa swimming forward in 80% of a straight line), sperm fast subpopulation (percentage of total cells), and cell concentration—were assessed with CASA using ISAS software (ISAS, Proiser R + D, S.L.); specified settings were adapted for fish species. For each sample (three fields per sample), a minimum of 200 spermatozoa were evaluated. When samples presented low concentrations, more than three fields were captured.

Total motility values obtained in day − 1 sampling were used to divide into homogeneous groups before the beginning of the CS protocol. Based on the data obtained by CASA, the individuals were arranged in one group or another until generating two populations that initially statistically had the same total motility mean values (same groups for trials 1 and 4).

The protocol was performed precisely one day after the ending of the CS protocol (day 22). Specific VIE tags in each male enabled individual tracking in each group and datasets comparison before and after stress induction.

### Statistics

We used the GraphPad Prism 9.0.0 package (GraphPad Software, Inc.) for statistical analysis and figure generation. All variables were tested for normal distribution using a Shapiro–Wilk normality test. When the two groups compared passed the normality test, significant differences between them were identified using a Student’s *t* test (paired or unpaired, as appropriate). For nonparametric data, a Mann–Whitney test was run. All data are shown as mean ± SEM (*p < 0.0500; **p < 0.0100; ***p < 0.0010; ns: no significant changes).

## Results

### Behavioral analysis

In the NTT, the 21-day CS protocol produced significant treatment effects (Fig. 2). There was no difference between control and CS fish (*p* > 0.0500) in kinetic parameters (velocity and swum distance ). Fish mean velocity remained close to 6 cm/s, and reported distance swam was 1750 cm (Fig. 2C). However, the experimental groups presented significant different swimming patterns depending on swimming zone preference (Fig. 2D). While staying in the lower zone—known as homebase because fish are released there at the beginning of the test—is a common tendency in all fish studied (Fig. 2D), fish in the S^−^ group spent significantly more time in the upper zone during the NTT (*p* < 0.0500) (Fig. 2D and E). These results from trial 1 validated the 21-day chronic-stress-induction protocol, as a modulation of behavior was observed when comparing groups.

### qPCR analysis of ER stress-related genes

qPCR results revealed that expression of both *bip* (*p* = 0.0494*)* and *chop* (*p* = 0.0317) genes were upregulated in the brains of animals exposed to the 21-day CS protocol (S^+^) compared with those in the control group (S^−^), as shown in Fig. 2F.

### Chronic stress does not alter tissular organization in zebrafish testes

**Fig. 3:**
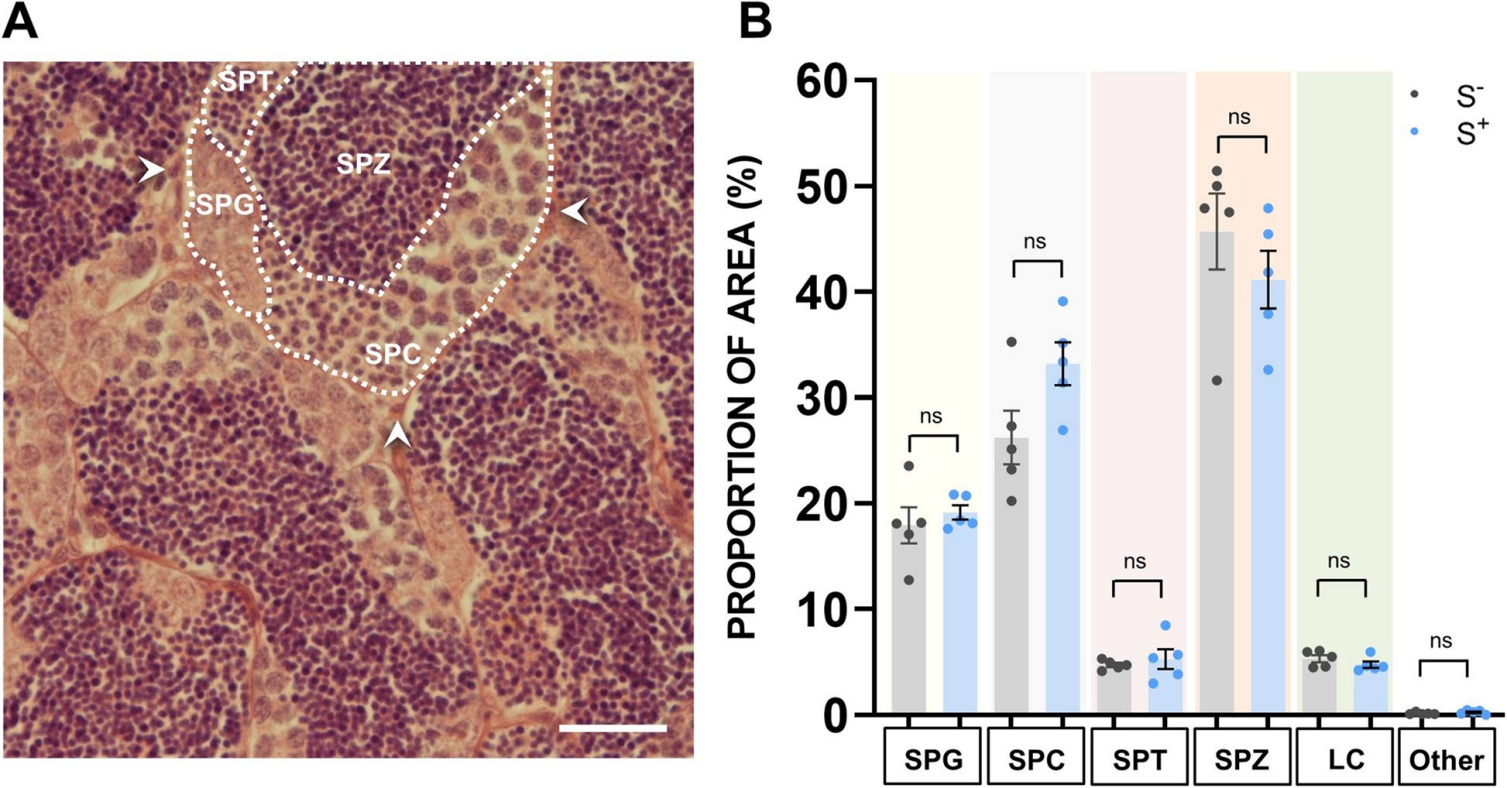
Histological analysis. **A** Representative H&E histological field of testes sections from S^−^ and S^+^. SPG, spermatogonia; SPC, spermatocytes; SPT, spermatids; SPZ, spermatozoa; LC, Leydig cells; Other, other cell types. Scale bar: 25 μm. **B** Quantitative analysis of spermatogenesis. Proportion (%) of area occupied by cells in S^−^ and S^+^. S^−^: control males. S^+^: males exposed to the CS protocol. Data are presented as mean ± SEM (*n* = 5; fish batch 2)

According to the histological quantification (Fig. 3), no noticeable architecture remodeling was found in testes of stressed males. None of the testicular cell populations showed statistically significant differences after the CS protocol (Fig. 5B). These results might suggest the presence of nondetectable alterations due to changes in testicular development derived from chronic stress exposure.

### Chronic stress downregulation of key genes in NMD pathway in testis

**Fig. 4:**
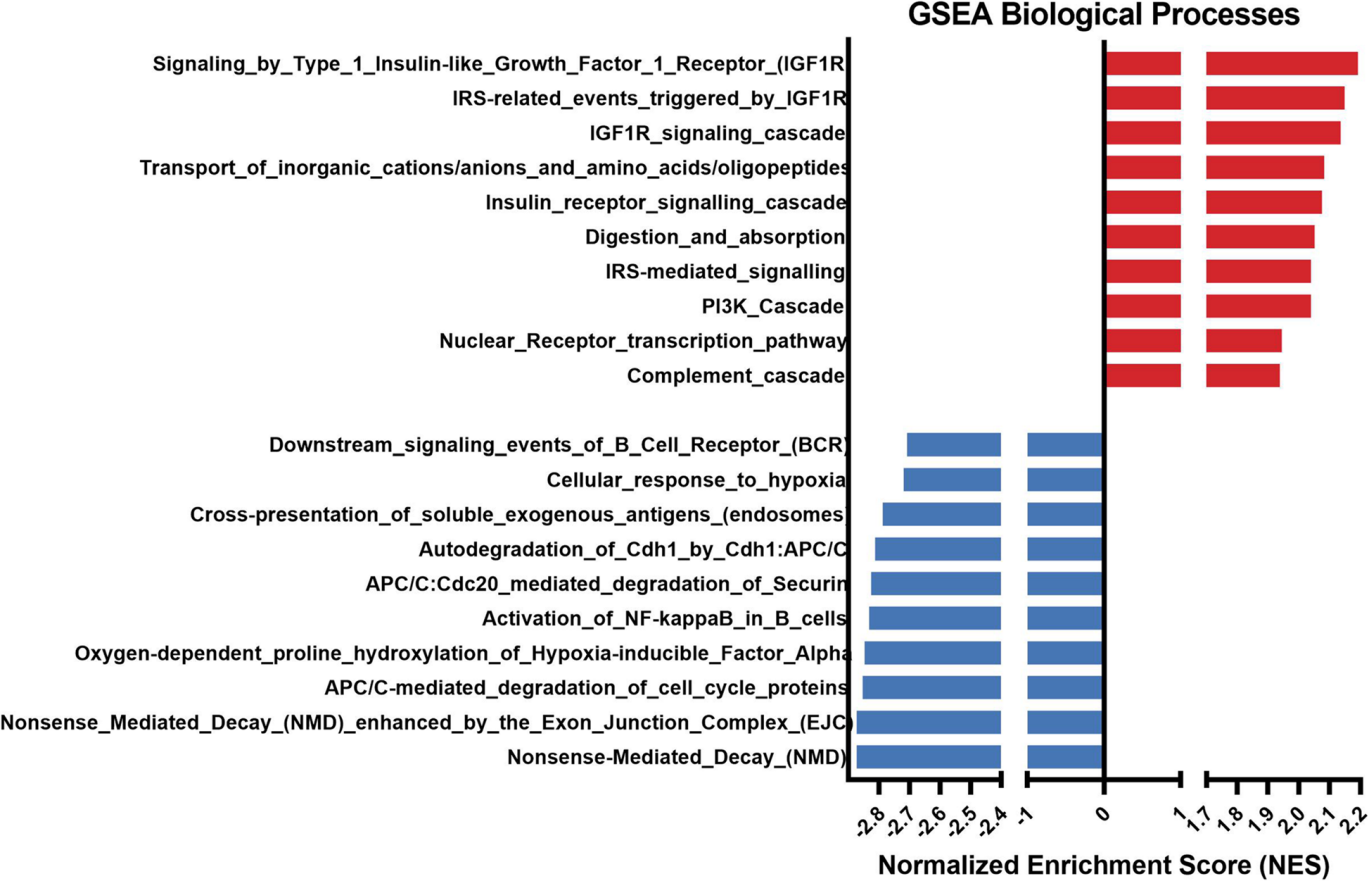
GSEA enrichment analysis in adult zebrafish testis tissue from males exposed to the CS protocol (fish batch 3). Normalized Enrichment Score (NES) was applied to correct differences in enrichment score between gene-sets due to differences in gene-set size

**Fig. 5:**
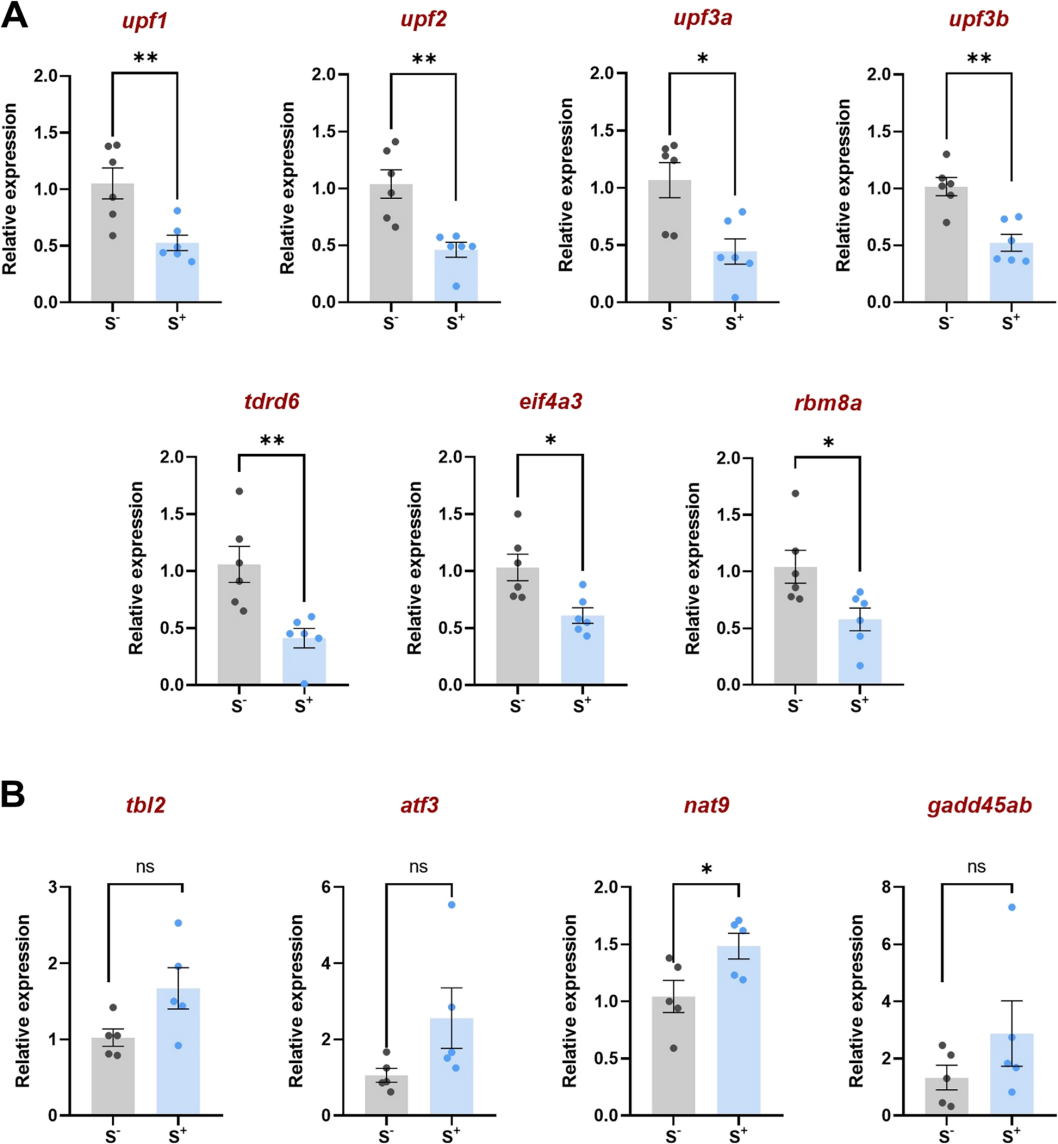
Relative gene expression in zebrafish testes of **A** Seven players involved in the nonsense mediated decay (NMD) pathway and **B** four substrates of the NMD pathway. S^−^: control males. S^+^: males exposed to the CS protocol. Data are presented as mean ± SEM (*n* = 5–6; batch 2). *p < 0.0500, **p < 0.0100

We compared gene expression profiles in testes from 3 CS male against 4 control male samples in our RNA-seq differential expression analysis. As an exploratory analysis, we performed a principal component analysis (PCA) using the top 500 most variable genes. Since the first two principal components did not show a clear separation of the two groups, unknown latent factors were removed with surrogate variable analysis (sva) coupled with limma-voom for differential expression testing. No genes passed the multiple test correction; however, after ranking the genes by the limma moderated *t*-statistic and running the Preranked GSEA module, the NMD pathway was enriched scoring the lowest negative normalized enrichment score (NES) values. Functionally, the pathways that scored the largest negative absolute value (NES = − 2.87) were the nonsense mediated decay (NMD) enhanced by the exon junction complex (EJC) and the nonsense mediated decay (Fig. 4). Genes belonging to this pathway were selected for orthogonal validation with qPCR. We focus our attention on the detailed study of these two pathways in qPCR studies in the testis.qPCR results showed that expression of all genes studied was significantly downregulated in the testicles of males exposed to the 21-day CS protocol (S^+^) compared with those in the control group (S^−^), as shown in Fig. 5. All genes coding for Up-frameshift proteins (UPFs) showed a significant downregulation close to a 0.5-fold decrease (*upf1*: *p* = 0.0063; *upf2*: *p* = 0.0022; *upf3a*: *p* = 0.0238; *upf3b*: *p* = 0.0011). Similarly, the gene coding for the Tudor domain-containing protein (*tdrd6*) showed a lower relative expression in the experimental group S^+^ (*p* = 0.0051). Statistically significant differences revealing downregulation in the stressed males were also found in the genes coding for the exon-junction complex (EJC) core proteins (*eif4a3*: *p* = 0.0248; *rmb8a*: *p* = 0.0112). Within the set of the four NMD substrates studied (*tbl2*, *atf3*, *nat9*, and *gadd45ab*), all the data showed a trend on overexpression in the S^+^ testicular samples although only the gene *nat9* showed significant differences between the two experimental groups (*p* = 0.0401).

### Chronic stress effect on quality of sperm in terms of motility

**Fig. 6:**
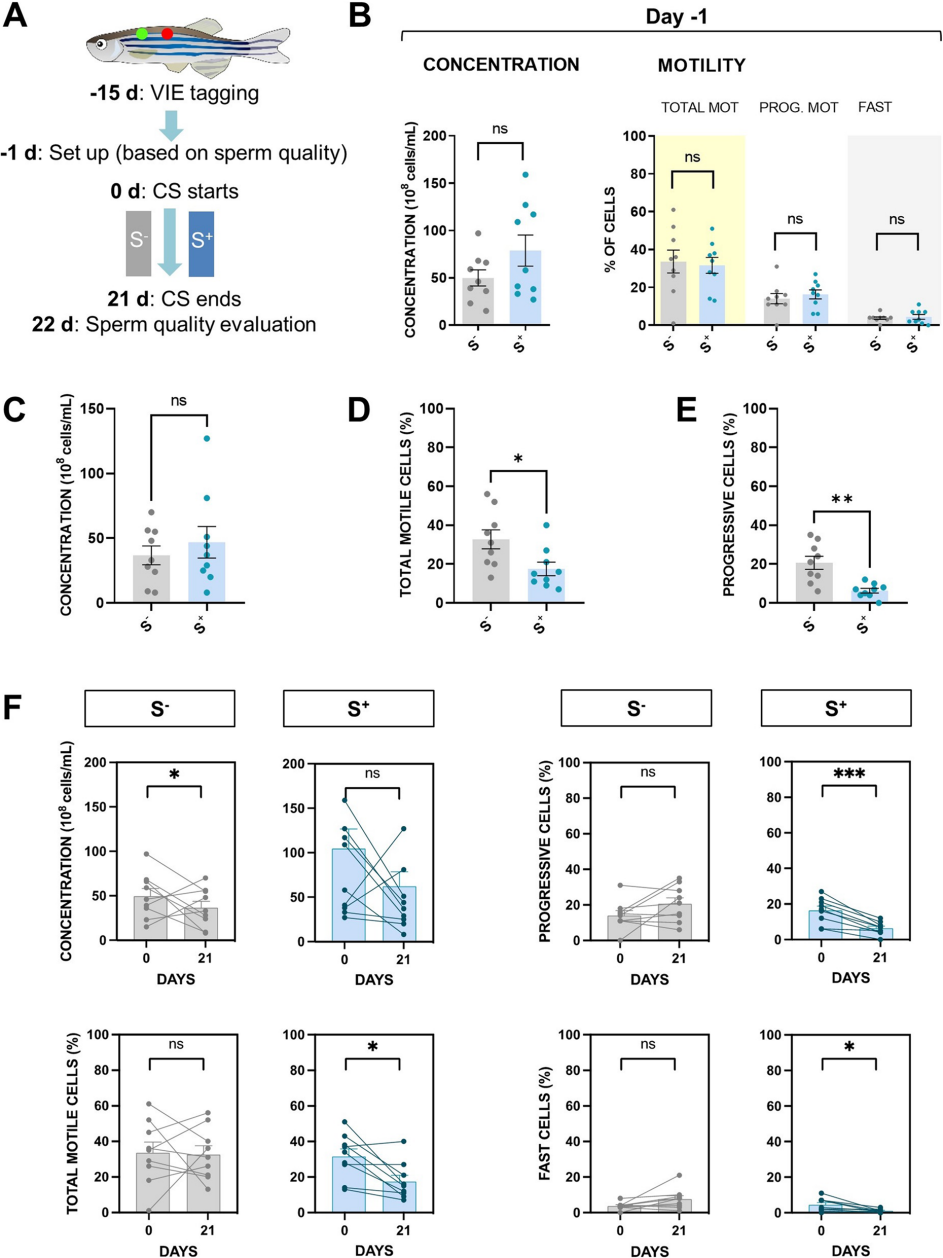
CS effects on zebrafish males in terms of sperm quality after approximately three rounds of spermatogenesis exposure. **A** Trial 4 design summary. Males were individually tagged 15 days before the beginning of the trial using visible implant elastomers (VIE). One day before the beginning of the CS protocol, males were divided into in homogeneous groups in terms of sperm quality parameters. After the 21-day CS protocol, on day 22, sperm samples were processed to evaluate the effect of chronic stress in the S^+^ group. **B** Sperm concentration, total motility, progressive motility, and fast cells fraction of the groups at day − 1. Mean values of **C** cell concentration, **D** total motility (%), and **E** progressive motility (%) after the trial 4 conclusion. **F** Before–after graphs for the experimental groups S^−^ and S^+^ for the endpoints concentration, total motility, progressive motility, and fast cell subpopulation. S^−^: control males. S^+^: males exposed to the CS protocol. Data are presented as mean ± SEM (*n* = 9; fish batch 1). *p < 0.0500, **p < 0.0100, ***p < 0.0010, ns, not significant (*p* > 0.0500)

To study the effects of chronic stress on spermatogenesis, we analyzed the concentration, total motility, and progressive motility of each sperm sample individually—VIE-tagging male tracking (Fig. 6A). At day − 1 in trial 3 (Fig. 6A), all males were split into two homogeneous groups according to a wide range of sperm motility values. Results regarding sperm concentration, total motility, and progressive motility in the group setting point (day − 1) are shown in Fig. 6B.

Exposure to chronic stress did not modify sperm count mean values when groups were compared after 21 days of the experiment (Fig. 6C). Males in the nonstressed control group showed a higher mean value (*p* = 0.0218) of total motility (%; mean ± SEM) compared with males in the stressed group (S^−^ = 32.78 ± 4.878 vs. S^+^ = 17.56 ± 3.481; Fig. 6D). Males in the stressed group showed a significantly lower mean value (*p* = 0.0011) on progressive motile cells percentage (%; mean ± SEM) compared with males in the nonstressed control group (S^+^ = 6.333 ± 1.190 vs. S^−^ = 20.67 ± 3.391; Fig. 6E).

The results of the before–after analysis (Fig. 6F) confirmed the deleterious effect of chronic stress on sperm quality. A statistically significant reduction trend was observed in the S^+^ group in total motile cells (%; *p* = 0.0122), progressive cells (%; *p* = 0.0003), and fast cells (%; *p* = 0.0211). These significant reductions observed in the paired Student’s *t* test were not found in the S^−^ group. However, the S^−^ group showed a reduction in cell concentration after 21 days (*p* < 0.0500).

### Larvae from males exposed to chronic stress presented an altered molecular signature

**Fig. 7:**
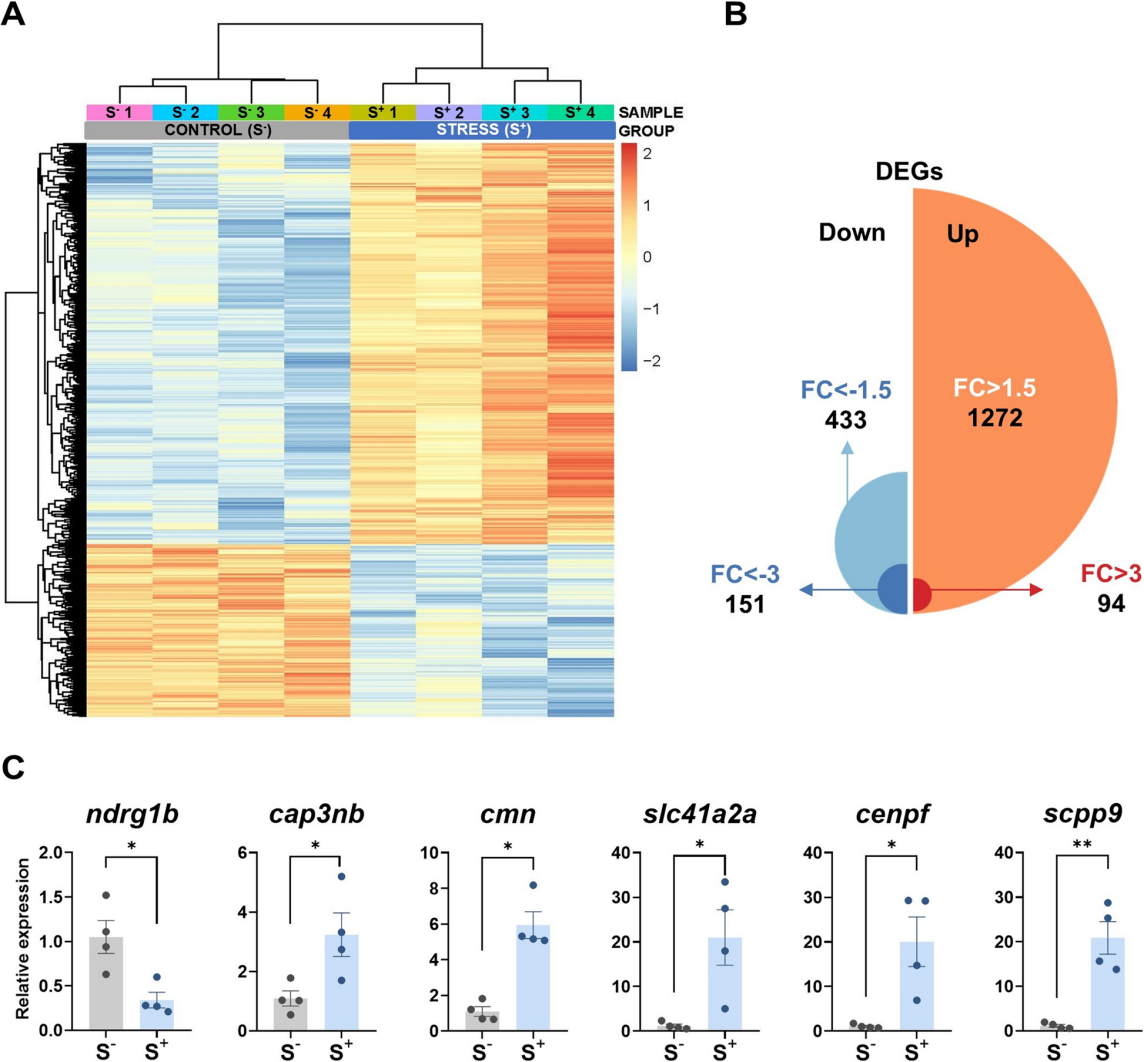
RNA-seq analysis of zebrafish 7-dpf larvae (control and stressed). Stressed progenies (S^+^) were obtained from a control female crossed with a male exposed to the 21-day CS protocol. Control progenies (S^−^) derived from a control unexposed male crossed with a control female. **A** Heatmap generated by unsupervised hierarchical clustering of RNAseq expression z-scores computed for the 1705 differentially expressed genes (DEGs) (cut-off criteria: *p*. adjusted < 0.0500; |FC| > 1.5) between S^−^ and S^+^ larvae. The heatmap was generated with the “pheatmap” R package (https://CRAN.R-project.org/package=pheatmap) (*n* = 4 progenies/treatment). **B** Representation of the 1705 DEGs (S^+^ vs S^−^) detected by RNA-seq passing the stablished threshold identifying the number of upregulated and downregulated DEGs and classifying them by their FC. **C** Normalized gene expression obtained in the qPCR validation assay for the RNA-seq. Data in **C** are shown as mean ± SEM (*p < 0.0500; **p < 0.0100)

In total, 20803 genes were detected. PCA of the expression data (Additional file 1: Fig. SM2) and heat map (Fig. 7A) showed that control and stressed-male derived progenies were clustered in two relatively well-separated groups, suggesting that the stressful conditions of male progenitors have a large effect on gene expression of the F1 individuals. RNA-seq revealed 1705 statistically significant (*p* adjusted < 0.0500) differentially expressed genes with a fold change |FC| > 1.5, 1272 of them upregulated in the S^+^ progenies and 433 downregulated (Fig. 7B; the complete list of these DEGs can be found in Additional file 5: Table 2, sheets 1 and 2). To facilitate data exploration, we established a cut-off value of |FC| > 3. This threshold reduced the number of genes to explore up to 245 differentially expressed transcripts (Fig. 7B; Additional file 5: Table 2, sheet 3); 151 of these DEGs were downregulated and 94 were upregulated. qPCR analysis of 6 DEGs (1 downregulated DEG in RNA-seq analysis: *ndrg1b*; 5 upregulated in RNA-seq analysis: *cpa3nb*, *cmn*, *slc41a2a*, *cenpf*, and *scpp9*) validated the sequencing results (Fig. 7C). Correlation between RNA-seq FC values and qPCR values for these set of genes can be found in Additional file 1: Figure SM3. We selected the top 245 DEGs and performed g:Profile analysis to study the enriched biological processes involving these genes showing a highly altered expression pattern between the experimental groups. The analysis reported a list of 29 biological processes, being the top ones entries linked to cell cycle (GO:1903047, GO:0022402, GO:0000278, GO:0007049), chromosome segregation (GO:0007059), and organization (GO:0051276) and DNA replication (GO:0006270) (Additional file 5: Table 2, sheet 4). Interestingly, g:Profile analysis for human phenotype ontology (Human Protein Atlas database) with the resulting enriched GO biological processes obtained from this batch of 245 top differentially expressed genes revealed that a relevant number of the entries were related to reproduction or fertility issues within the 15 top positions of the list: absent testis (HP:0010469), decreased fertility in males (HP:0012041), abnormality of the female genitalia (HP:0010460), abnormal male reproductive system physiology (HP:0012874), or functional abnormality of male internal genitalia (HP:0000025). The list of human phenotypes also included in the top positions entries related to musculoskeletal system: clubbing (HP:0001217), sloping forehead (HP:0000340), and clubbing of toes (HP:0100760); intestinal stenosis: small intestinal stenosis (HP:0012848) and duodenal stenosis (HP:0100867); and chromosome stability entries: abnormality of chromosome stability (HP:0003220) and abnormality of chromosome stability (HP:0003220). The complete list of the HP entries is included in Additional file 5: Table 2, sheet 5.

**Fig. 8:**
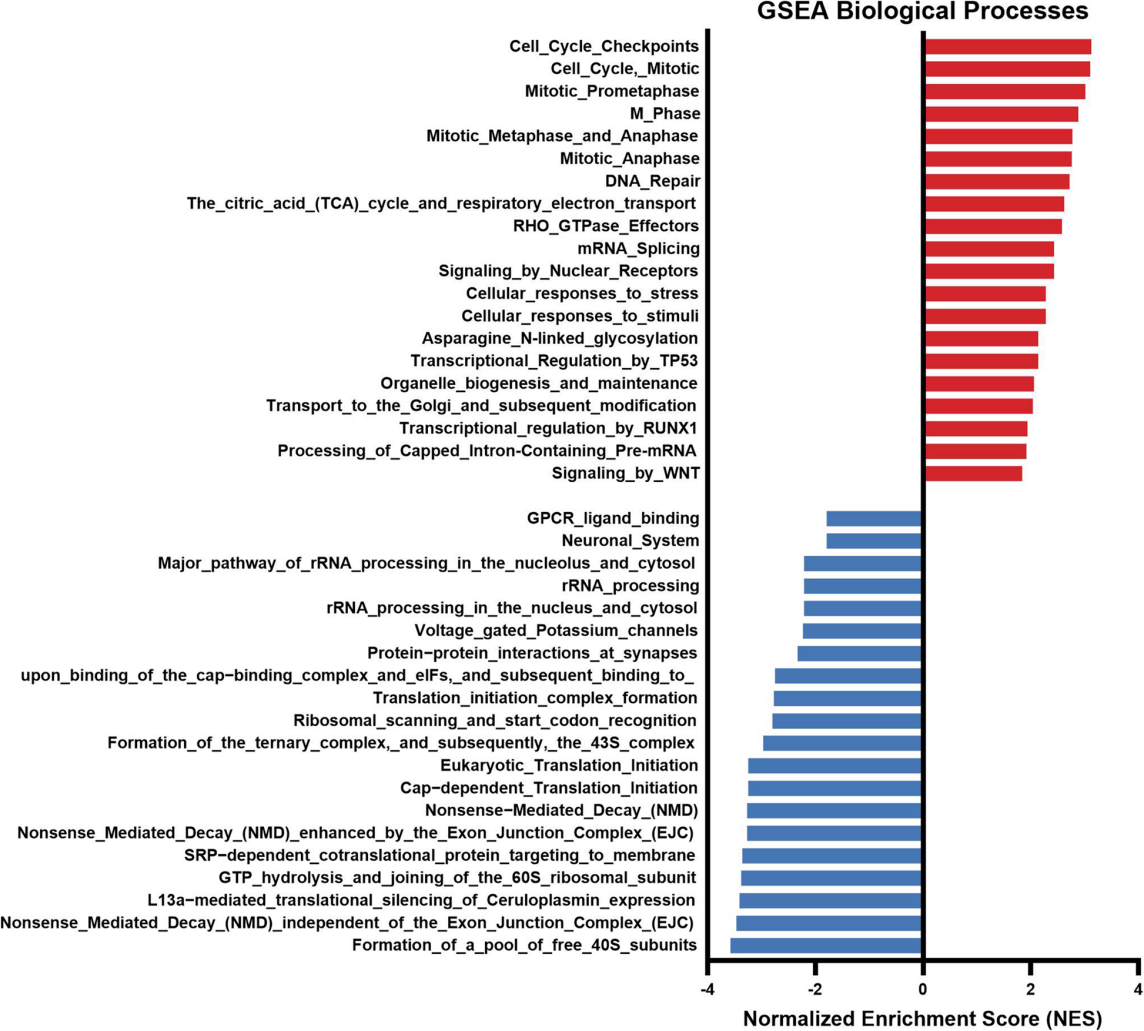
GSEA enrichment analysis considering biological processes of RNA-seq in zebrafish 7 dpf S^+^ progenies (derived from males exposed to the chronic stress protocol). Normalized enrichment score (NES) was applied to correct differences in enrichment score between gene-sets due to differences in gene-set size

In order to get gene sets sharing common biological processes, GSEA analysis was performed using bulk data from RNA-seq data from 7 dpf larvae in order to facilitate comparison with the GSEA performed on the testis data. Our results highlighted several biological processes affected on the progenies after male stress induction. The GSEA analysis revealed that the samples of stressed group were enriched in “cellular responses to stress” and “cell response to stimuli,” both of them associated to stress response. Cell cycle, mitosis, and DNA repair represented the biological processes with the highest enrichments (NES > 2.5) (Fig. 8). Contrary to this, mRNA translation and their associated pathways were downregulated in zebrafish larvae form stressed males. Moreover, according to our previous results in the gonads of males exposed to CS protocol, some biological processed related to nonsense-mediated decay (NMD) and NMD independent of the exon junction complex (EJC) scored the largest negative absolute values (NES > − 3.2) (Fig. 8).

## Discussion

In a stressful situation, the organism responds by changing physiological processes in an effort to reestablish homeostasis, a complex dynamic equilibrium [6, 8]. These stress-derived alterations are multiple and diverse and usually accompanied by biochemical and behavioral changes or responses [6, 8]. The potential role of chronic stress as a promotor of infertility as a response to homeostasis disruption has been previously suggested, mainly based on clinical and cross-sectional studies [5–7]. In the present work, we used zebrafish as a powerful vertebrate model to integrate physiological responses with molecular modulations to shed light on the effect that chronic stress induction can have on male gametogenesis. Our in vivo data demonstrate that a chronic physiological stress induction protocol encompassing around 3 waves of spermatogenesis cycle can significantly affect this process, causing alterations in key regulatory pathways. The data presented herein strongly supports the hypothesis that a stressful environment has a direct impact on male reproduction [7], since our results show a reduction in the quality of the resulting sperm. Thus, our study is an extensive characterization of the effects of chronic stress on male reproduction in zebrafish.

Fish subjected to the chronic stress induction protocol showed a swimming pattern typical of animals with high anxiety levels. These individuals were characterized by their geotaxis in the NTT experiment compared with the ones in the control group (Fig. 2), whose swimming pattern included greater time in the upper zone of the experimental tank. These results are in line with what has been previously described in stress evaluation experiments with zebrafish [28, 44, 45].

In the zebrafish testicle, spermatogenesis lasts around 6 days, as has been previously described [27]. Together with the results reported by behavioral studies, our qPCR experiments confirmed the impact at a molecular level of the 21-day stress (involving around 3 cycles of spermatogenesis) protocol caused an activation of two genes that encode for the protein chaperone BIP and the transcription factor CHOP. The accumulation of these two messenger RNAs is considered a result of ER stress [46]. Among the cellular alterations observed as a consequence of environmental stress, alterations to organelle function at ER level has been described as a factor contributing to neuronal dysfunction [47]. Our results validate the stress induction protocol for this temporal frame (Fig. 2), establishing solid foundations not only for the development of the rest of the trials included in this experimental design but also for future experiments focusing on correlating stress and reproduction.

Several studies have shown prolonged stress to cause morphological and functional alterations in testes; for example, in mammal models, several functional (testosterone level and sperm quality) and morphological (testicular weight and seminiferous tubular diameter) reductions after 6-week stress stimuli (immobilization method) have been shown [48]. In experiments involving teleost models, starvation-derived stress was found to alter testicular structure and cause spermatogenesis impairment reflected by an increased number of spermatogonia and spermatozoa together with a lower proportion of the spermatids population in the starved fish [49].

We performed histological experiments to explore whether alterations in cell populations occurred at tissue level in the gonads of the stressed specimens in the present study. No substantial impact on cell-type populations was observed (Fig. 3). Although data do not show significant tissue remodeling, our results point to a trend indicating that chronic stress may slightly modulate the proportion of spermatocytes and spermatozoa, which could be an indication of a hypothetical spermatogenesis arrestment. It could be possible that the disruption to spermatogenesis may be driven by more subtle molecular changes rather than to cell population proportion modification.

We considered it highly relevant to identify the gonadal molecular targets involved in a chronic stress situation and to integrate them in the molecular networks that orchestrate gametogenesis. For this purpose, we performed a GSEA of the RNA-seq results in the zebrafish testicles. Our results (Fig. 4) highlighted several biological processes affected after stress induction. We focused on the NMD pathway, which was clearly deregulated in stressed males, scoring the lowest negative normalized enrichment score (NES) values. NMD is a translation-coupled mechanism that eliminates mRNAs containing premature translation-termination codons (PTCs) and regulates the abundance of a large number of cellular RNAs [50, 51]. NMD is suppressed by diverse stressors [51], such as infection, nutrient deprivation, or hypoxia [52, 53], and is crucial in spermatogenesis [54]. This NMD inhibition is partially triggered by the phosphorylation of the translation initiation factor eIF2, which leads to the inhibition of mRNA translation; this represents a beginning stage of numerous stress pathways [55]. Previous studies investigating spermatogenesis in mammalian models [56, 57] have shown that NMD operates by shaping the male-germ-cell-specific transcriptome (characterized by mRNAs with unusually short 3′UTRs [58]) clearing ubiquitously expressed mRNAs with long 3-UTRs (via UPF2- and TDRD6-dependent degradation).

In view of the results from our GSEA, we decided to focus on seven key genes in the NMD pathway (Fig. 5): UPF1 RNA helicase and ATPase (*upf1*), UPF2, UPF3A, and UPF3B regulators of NMD (*upf2*,* upf3a*, and *upf3b*), Tudor domain-containing protein (*tdrd6*), eukaryotic initiation factor 4E (*eif4a3*), and RNA-binding motif protein 8A (*rmb8a*). *upf1*, *upf2*, *upf3a*, and *upf3b* are evolutionarily conserved genes coding for transacting factors in NMD [50]. These three UPF proteins (UPF2, UPF3A, and UPF3B) have been shown to form the core complex of the NMD machinery, which leads to premature translation termination and therefore to mRNA degradation [59]. In mammalian models, postmeiotic spermatocytes and spermatids highly express UPF1 and UPF2 [56, 57], which are located in the chromatin bodies (CBs), large germ-cell-specific perinuclear structures which appear during the meiotic/postmeiotic switch in spermatogenesis [60, 61]. CBs play a role in Piwi-interacting RNA (piRNA) biogenesis and contain RNA and proteins such as RNA-binding proteins, helicases, and several members of the Tudor-domain protein family (TDRDs) such as TDRD6. TDRD6 knockout studies have revealed that this protein is required for localization of NMD degradation machinery to the CB [57]. Mutations on this gene are associated to oligoasthenozoospermia in humans [62], and expression of genes coding for TDRDs is very low in azoospermic men [63].

In the present work, we also studied the factors eIF4A3 and RBM8A (also known as Y14), both part of the exon junction complex (EJC), a multisubunit protein complex, which is placed 20–24 nucleotides upstream of an exon–exon which serves as an anchor point for NMD factor [64]. Consistently with the GSEA of the RNA-seq results, our qPCR experiments resulted in significant decreased expression of the selected seven key genes involved in the NMD pathway. Our results are in good agreement with previous studies investigating the link between NMD dysregulation and gametogenesis in model species; for example, ablation of *upf2* in murine embryonic Sertoli cells (SCs) leads to severe testicular atrophy and male sterility owing to rapid depletion of both SCs and germ cells [56]. The dysregulation or malfunction of this pathway is significantly negative and can have important consequences in the testicular cells; for example, in infertile men showing mutations in *CATSPER* gene, the resulting aberrant transcripts translate into severely truncated proteins if these are not subjected to NMD [65].

The study of the gene expression of the well-characterized human endogenous NMD substrates ATF3, GADD45B, NAT9, and TBL2 [39] reported a different pattern, showing higher expression values in the S^+^ group, being significant only for *nat9* (Fig. 4B). Thus, it cannot be concluded that there is a strong or total suppression of the NMD pathway under stress conditions. However, these results are logical in line with the reported downregulation of the NMD factors in the samples and are in line with previous studies where the depletion of NMD factors by RNAi provoked the overexpression of these genes coding for the NMD substrates [39].

With our fourth trial, we sought to determine the effect of stress on the final gamete. To evaluate a few waves of spermatogenesis, we performed an experiment with individually identified specimens from whom sperm was extracted just before the start of the stress induction. We performed this before-after CS approach since the studied sperm parameters are subject to temporal fluctuations as it can be seen in the S^−^ group in which we registered a significant reduction in cell concentration after 21 days (Fig. 6). In our examination of CS-exposed individuals, we found a clear reduction in sperm quality. We used motility as a variable to measure gamete quality; this has been described as a tool for prediction of in vitro fertilization success in humans [66]. Sperm motility in specimens exposed to stress during spermatogenesis was significantly reduced in total and progressive values (Fig. 6). Moreover, the number of fast cells (with a higher probability of successful fertilization [67]) was also reduced. These results are relevant since these reveal the link between dysregulation at molecular level in the NMD regulation pathway and final sperm quality. In line with our results, deletion of Tex13a—a spermatid-specific partner of CCR4–NOT complex reported to participate in the mRNA degradation in the late spermatids—has been recently shown to lower sperm motility in murine models [68]. Despite key anatomical differences between the mammalian HPA and teleost HPI axes, core elements of glucocorticoid signaling pathways are highly conserved. Furthermore, the zebrafish has been suggested as a promising model for vertebrate spermatogenesis as molecular markers of sperm quality are shared between humans and zebrafish [69]; therefore, we cannot discard this possibility, and we consider that our findings can contribute to a better understanding of the effects of stress in vertebrates.

To elucidate the effects that a parental trauma can have in the offspring is very complex and several investigations have been mainly focused in behavioral alterations, emotional issues, and mental adverse outcome such as depression and anxiety disorders [70], although physical health problems such as diabetes, hypertension, obesity, cardiovascular disease, and increased susceptibility to infections among others have been also reported [71]. In recent years, molecular studies have provided new information that could shed light on this complex issue. As an example, RNA-seq analysis of the amygdala of mice derived from fathers exposed to chronic unpredictable stress discovered alterations in several pathways, being the most affected the “notch signaling” one [72]. Again, for these molecular studies, species such as mice or zebrafish are excellent models [73]. In the last approach of this work, we perform transcriptomic analyses in the resulting progenies derived from exposed males crossed with unexposed females, to identify molecular mechanisms putatively compromised by paternal exposure to chronic stress. Our GSEA analysis in the progeny trial, revealed an upregulation of some pathways related to stress: “cellular responses to stress” and “cellular responses to stimuli” in stressed larvae strongly supporting a stress progeny transmission of male exposed to CS protocol (Fig. 8). A high percentage of enriched biological processes in unexposed larvae from stressed parents were involved in DNA repair, cell cycle control, and different mitosis phases presenting the highest normalized enrichment score (NES) values (Fig. 8). Contributing to these enriched pathways, many of the top overexpressed genes (Supplementary Material Table 2) in the S^+^ larvae corresponded to the following: cyclins (cyclin B1, B2 (*ccnb1*, *ccnb2*)), cyclin kinases (*cdk1*) denticleless protein homolog (*dtl*), centromere associated proteins (*cenpf*, *cenph*,* incenp*), DNA replication licensing factors mini-chromosome-maintenance 2-7 proteins (*mcm2*,* mcm3*,* mcm5*,* mcm6*, and *mcmc7*), the transcription factor *e2f7*, MAD2 mitotic arrest deficient-like 1 (*mad2l1*), assembly factor for spindle microtubules aspm among others (*aspm*). Contrary to this, a downregulation of mRNA translation and their associated biological processes was observed. The attenuation of global mRNA translation contributing to induction of stress response proteins as a mechanism to counteract the induced molecular damage [74]. The cap-dependent translation is initiated by eIF4E (eukaryotic initiation factor 4E) binding to the end of mRNA through the cap 5′ structure. Under certain stress conditions, translation is drastically reduced inhibiting the eIF4E leading a reduction in global translation and subsequently activation of an alternative pathway to preserve selective proteins for stress response [75–77]. Our results are in line with these findings supporting again the potential vertical transmission of stress effects. Furthermore, a diminish of NMD pathways was observed in the progenies of stressed males, scoring low negative NES (Fig. 8). Thus, stressed-derived larvae showed some shared altered transcriptomic patterns that were pointed in parental testicular GSEA. The dysregulation (copy number alterations in NMD genes) of this pathway in humans is correlated with neurodevelopmental disorders, including schizophrenia and autism [78–82]. Interestingly, we found within the top 20 upregulated DEGs in the S^+^ larvae the gene *apobb*.2, codying for the apolipoprotein B (ApoB) which overexpression has been positively correlated to neurodegeneration, cognitive deficits, and depression in humans [82] and animal models [83]. Another top downregulated gene in the stress-derived larvae was linked to vitamin metabolic process, cytochrome P450, family 24, subfamily A, and polypeptide 1 (*cyp24a1*) involved in vitamin D metabolism. Vitamin D is a fat-soluble vitamin, which plays a key role in calcium and phosphate homeostasis [84]. Vitamin D deficiency has been suggested to be associated with an enhanced risk of major depressive disorder and anxiety disorders and therefore many therapies are based on vitamin D intake supplements [85].

In terms of canonical markers of stress response, 12 of de upregulated DEGs in the S^+^ larvae (Additional file 3: Table 2) belong to the heat shock protein family (Hsps): *dnajb11*, *dnajc11b*, *hspe1*, *hspd1*, *hspb6*, *hspa9*, *hspa5*, *hspa4l*, *hspa4a*, *hsp70*.3, *hsp90b1*, and *hsp90aa1.1*. Hsps are molecular chaperones that have been characterized for their roles in protein maturation, re-folding, and degradation [83]. In the presence of stressful stimuli, Hsps are rapidly upregulated [86]. Other stressed-related genes statistically overexpressed in S^+^ larvae in our experiment belong to the protein disulfide isomerase (PDI) family (DEGs: *pdia4*, *pdia5*, *pdia6*, and *pdia7*). Initially, the main function of the PDIs was defined to promote oxidative protein folding in the ER, but during the last years, other roles have been linked to this family such as ER-associated degradation (ERAD), trafficking, calcium homeostasis, antigen presentation, and virus entry [84]. Other well-stablished genes linked to stress in human and mammals [85] were altered in our experiment: proopiomelanocortin (*pomca)*, nuclear receptor subfamily 3 group C member 2 (*nr3c2*), melanocortin 1 receptor (*mc1r*) and purinergic receptor P2X, ligand-gated ion channel, 7 (*p2rx7*). These molecular alterations clearly point to an intergenerational paternal transmission of stress and shed light on stress effects under a molecular prism.

## Conclusions

Our results show that induction of chronic stress in the zebrafish model has different deleterious impacts on behavior and male reproduction. Chronic stress was associated with increased anxiety-like behavior and brain overexpression of genes involved in ER stress. Induction of chronic stress during spermatogenesis caused downregulation of the key NMD surveillance pathway in the gonads. Modulation of the gene expression pattern in the testes did not correlate to tissular changes, although stress-derived spermatozoa reported lower quality in terms of motility on the final gamete. The resulting progenies of stressed progenitors registered molecular alterations potentially compromising the offspring. Together, our results are paramount to increase the ever-expanding understanding of the effects of chronic stress on reproduction.

## Supplementary Information

**Additional file 1:** **Fig S1.** Validation ofvideo-based exposure to a zebrafish predator. **Fig S2.** Principal component analysis (PCA) of RNA-seq performed in7 dpf larvae. **Fig S3.** RNA-seq vsqPCR correlation values.

**Additional file 2:** **Script 1.** Script limma_sva.r.

**Additional file 3:** **Script 2.** Script fgsea.r.

**Additional file 4:** **Table 1.** Designed primersused in gene expression analyses by real-time qPCR.

**Additional file 5:** **Table 2.** Sheet 1-Downregulated DEGs. Sheet 2- Upregulated DEGs. Sheet 3- DEGs passing thecut-off value |FC|>3. Sheet 4- Biological Processes involving DEGs passingthe cut-off value |FC|>3. Sheet 5- Entries from the Human Phenotype databaseinvolving the Biological Processes reported by DEGs passing the cut-off value|FC|>3.

## Data Availability

All data generated or analyzed during this study are included in this article and its additional files. RNA-seq data can be found in https://identifiers.org/ncbi/geo:GSE212999 and https://identifiers.org/ncbi/geo:GSE224132.

## Abbreviations

CASA: Computer-assisted sperm analysis
CB: Chromatin bodies
CS: Chronic stress
DEGs: Differentially expressed genes
EJC: Exon junction complex
ER: Endoplasmic reticulum
ERAD: ER-associated degradation
FC: Fold change
GO: Gene ontology
GSEA: Gene set enrichment analysis
HBSS: Hank’s balanced salt solution
HKG: Housekeeping gene
HPA: Hypothalamic–pituitary–adrenal
HPG: Hypothalamic–pituitary–gonadal
HSP: Heat shock protein
H&E: Hematoxylin eosin
mRNA: Messenger RNA
NCBI: National Center for Biotechnology Information
NES: Normalized enrichment score
NMD: Nonsense-mediated decay
NTC: Not template control
NTT: Novel tank test
PBS: Phosphate-buffered saline
PCA: Principal component analysis
PDI: Protein disulfide isomerase
piRNA: Piwi-interacting RNA
PTC: Premature translation-termination codon
qPCR: Quantitative polymerase chain reaction
RIN: RNA integrity number
rRNA: Ribosomal RNA
RNA-seq: RNA sequencing
S^−^: “Control” samples
S^+^: “Stress” samples
SC: Sertoli cells
SSCs: Spermatogonial stem cells
SVA: Surrogate variable analysis
TAE: Tris-acetate-EDTA
TDRD: Tudor-domain protein family
UCS: Unpredictable chronic stress
VIE: Visible implant elastomers
