# Supplementary material for: Stress decreases spermatozoa quality and induces molecular alterations in zebrafish progeny
Source: BMC Biol. 2023 Apr 3;21:70. doi: 10.1186/s12915-023-01570-w (PMC10071778; doi:10.1186/s12915-023-01570-w)
Supplement: Supplementary file 1 — Additional file 1: Fig S1. Validation ofvideo-based exposure to a zebrafish predator. Fig S2. Principal component analysis (PCA) of RNA-seq performed in7 dpf larvae. Fig S3. RNA-seq vsqPCR correlation values. [file 12915_2023_1570_MOESM1_ESM.docx]

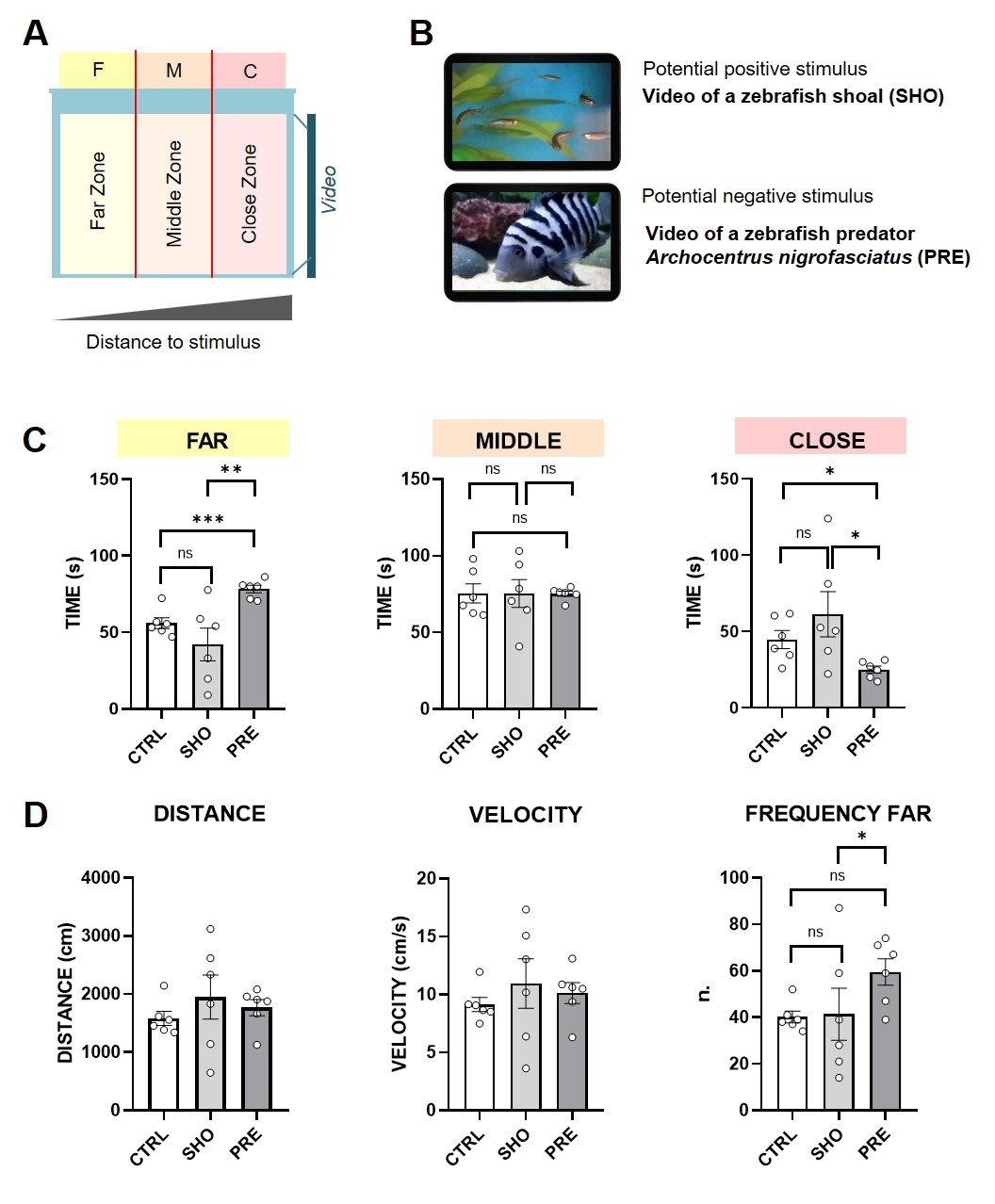


**Figure SM1** Validation of replacement of real exposure to a specimen of *Archocentrus nigrofasciatus* with a video of this species projected on one of the walls of the induction tank. **A** Virtual zones (close, middle, and far) included in evaluation arena established with Noldus Ethovision^®^ XT16 software on the proximity of the stimulus (video). NTT duration = 3 min. **B** Capture frames of videos used as potential positive stimulus (SHO) and potential negative stimulus (PRE). **C** Zone preference of each experimental group. **D** Kinetics parameters: distance, velocity and number of entries (n.) in the “far” zone of the tank. CTRL: control fish unexposed to stimulus. SHO: fish exposed to a video of a shoal of zebrafish. PRE: fish exposed to a video of a specimen of the zebrafish predator *A. nigrofasciatus*. Data are presented as mean ± SEM (*n* = 6; 3 males, 3 females). **p* < 0.0500, ***p* < 0.0100, ****p* < 0.0010, not signifcant (*p* > 0.0500).

**Figure SM2.** Principal component analysis (PCA) of RNA-seq using the top 500 most variable genes between control and stressed experimental groups (n = 4 pools per treatment of unexposed zebrafish 7-dpf larvae). Samples AU1024, AU1026, AU1027 and AU1028 (labelled in black) correspond to the control (S^-^) male-derived progenies and samples AU1035, AU1036, AU1037 and AU1038 (labelled in red) correspond to the chronically stressed (S+) male-derived progenies.

**
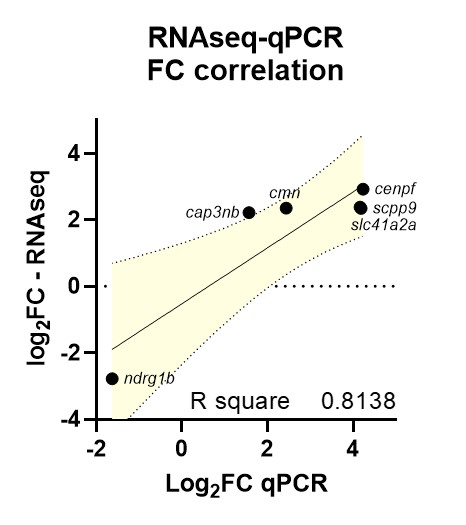
**

**Figure SM3.** RNA-seq vs qPCR correlation values for the validation of 7dpf larvae RNA-seq experiment.
