# Supplementary material for: Stress decreases spermatozoa quality and induces molecular alterations in zebrafish progeny
Source: BMC Biol. 2023 Apr 3;21:70. doi: 10.1186/s12915-023-01570-w (PMC10071778; doi:10.1186/s12915-023-01570-w)
Supplement: Supplementary file 2 — Additional file 2: Script 1. Script limma_sva.r. [file 12915_2023_1570_MOESM2_ESM.docx]

#!/usr/bin/envRscript

# author: Anna Esteve Codina <anna.esteve@cnag.crg.eu>

library(edgeR)

library(limma)

library(sva)

#info=read.table("info_roblesvan_01_gonad_male_only",h=T,row.names=1)

counts=read.table("COUNTS_genes_ROBLESVAN_01",h=T,row.names=1)

counts=counts[,colnames(counts) %in% rownames(info)]

info=info[colnames(counts),]

y=DGEList(counts=counts)

A<-rowSums(y$counts)

isexpr<-A>500

y=y[isexpr,keep.lib.size=FALSE]

dim(y)

y=calcNormFactors(y)

group=factor(info$GROUP)

mod <- model.matrix(~group, info)

mod0 <- model.matrix(~1, info)

v=voom(y,mod)

n.sv = num.sv(v$E,mod)

#n.sv=2

sva_obj <- sva(v$E, mod, mod0, n.sv=n.sv)

mod1 <- model.matrix(~group+sva_obj$sv)

colnames(mod1)=c("Intercept","groupUnaffected","SV1","SV2")

v <- voom(counts=y, design = mod1)

contr.matrix <- makeContrasts(

Affected_vs_Unaffected=-groupUnaffected,levels=mod1)

fit=lmFit(v,mod1)

fit=contrasts.fit(fit, contrasts=contr.matrix)

fit2=eBayes(fit)

summary(decideTests(fit2))

top=topTable(fit2, coef=1, sort="p", n=Inf)

write.table(top,"ROBLESVAN_01_limmavoom_sva_results_gonad.txt",quote=F)

write.table(v$E,"ROBLESVAN_01_limmavoom_expression_gonad.txt",quote=F)
