# Supplementary material for: Stress decreases spermatozoa quality and induces molecular alterations in zebrafish progeny
Source: BMC Biol. 2023 Apr 3;21:70. doi: 10.1186/s12915-023-01570-w (PMC10071778; doi:10.1186/s12915-023-01570-w)
Supplement: Supplementary file 3 — Additional file 3: Script 2. Script fgsea.r. [file 12915_2023_1570_MOESM3_ESM.docx]

| #!/usr/bin/envRscript |  |
| --- | --- |
|  |  |
|  | # author: Marc Dabad <marc.dabad@cnag.crg.cat> |
|  | # author: Beatriz Martin-Mur <beatriz.martin@cnag.crg.eu> |
|  |  |
|  | ### Parse args |
|  |  |
|  | args = commandArgs(trailingOnly=TRUE) |
|  | usage <- function(){ |
|  | cat(" |
|  | The R Script |
|  |  |
|  | Arguments: |
|  | --input=file - Pattern file name [mandatory] |
|  | --outdir=path - Path to output_dir [default: ./] |
|  | --gmt=file - Path to gmt file [mandatory] |
|  | --split=char - String to split name (first column) [default: \"\\.\"] |
|  | --colid=int - From first column, where is the ID to be used (gene_name, gene_id...) (default: 1) |
|  | --rank=int - Column to rank [mandatory] |
|  | --help - print this text |
|  |  |
|  | Example: |
|  | ./fgsea.R --input=pattern \\ |
|  | --ouputdir=\"path/to/output\" \\ |
|  | --gmt=\"path/to/file.gmt\" \\ |
|  | --split=\".\" \\ |
|  | --rank=4\n\n") |
|  |  |
|  | q(save="no") |
|  | } |
|  |  |
|  | ## Default setting when no arguments passed |
|  | if(length(args) < 1 \|\| args == "--help") { |
|  | usage() |
|  | } |
|  |  |
|  |  |
|  | ## Parse arguments (we expect the form --arg=value) |
|  | parseArgs <- function(x) strsplit(sub("^--", "", x), "=") |
|  | argsDF <- as.data.frame(do.call("rbind", parseArgs(args))) |
|  | opt <- as.list(as.character(argsDF$V2)) |
|  | names(opt) <- argsDF$V1 |
|  |  |
|  |  |
|  |  |
|  | if(is.null(opt$input)){ |
|  | cat("ERROR: --input is a mandatory fields") |
|  | usage() |
|  | } |
|  | if(is.null(opt$outdir)){ |
|  | opt$outdir<-"./" |
|  | } |
|  |  |
|  | if(is.null(opt$colid)){ |
|  | opt$colid<-1 |
|  | }else{ |
|  | opt$colid<- as.numeric(opt$colid) |
|  | } |
|  |  |
|  | if(is.null(opt$gmt)){ |
|  | cat("ERROR: --gmt is a mandatory fields") |
|  | usage() |
|  | } |
|  |  |
|  | if(is.null(opt$split)){ |
|  | opt$split<-"\\." |
|  | }else{ |
|  | if (opt$split=="."){ |
|  | opt$split<-"\\." |
|  | } |
|  | } |
|  |  |
|  | if(is.null(opt$rank)){ |
|  | cat("ERROR: --rank is a mandatory fields") |
|  | usage() |
|  | } |
|  |  |
|  | print(opt) |
|  |  |
|  |  |
|  |  |
|  |  |
|  | ### LIBRARIES |
|  | library('fgsea') |
|  | library("repr") |
|  | library('data.table') |
|  |  |
|  |  |
|  | ### FUNCTIONS |
|  | run_fgsea <- function(fx, sepName, rankINT, allLevels){ |
|  | bname = tools::file_path_sans_ext(basename(fx)) |
|  | raw_table <- read.table(fx, sep=" ", header=T) |
|  | rankName <- colnames(raw_table)[rankINT] |
|  | print(paste("Ranking by: ", rankName, sep="")) |
|  | ranks <- data.frame(list(ID=sapply(strsplit(rownames(raw_table), |
|  | sepName), |
|  | function(x) unlist(x)[opt$colid]), |
|  | shrunkenlfc=raw_table[,rankINT])) |
|  | ranks<- unique(ranks) |
|  | ranks <- setNames(ranks$shrunkenlfc, ranks$ID) |
|  | ranks <- ranks[!is.na(ranks)] |
|  | fgseaRes <- fgsea(allLevels, ranks, nperm=1000000, maxSize=500) |
|  | pdf(paste(bname,"_fgsea.pdf", sep=""), width = 15, height = 10) |
|  | topPathwaysUp <- fgseaRes[ES > 0][head(order(pval), n=20), pathway] |
|  | topPathwaysDown <- fgseaRes[ES < 0][head(order(pval), n=20), pathway] |
|  | topPathways <- c(topPathwaysUp, rev(topPathwaysDown)) |
|  | plotGseaTable(allLevels[topPathways], ranks, fgseaRes, |
|  | gseaParam = 0.5, colwidths = c(5, 3,0.8, 1.2, 1.2)) |
|  | dev.off() |
|  | fwrite(fgseaRes[padj<0.05][order(pval,NES)], |
|  | file = paste(bname,"_fgsea.tsv", sep = ""), |
|  | sep = "\t", |
|  | sep2 = c("", " ", "")) |
|  | } |
|  |  |
|  |  |
|  | ### MAIN |
|  | files <- Sys.glob(opt$input) |
|  | allLevels <- gmtPathways(opt$gmt) |
|  | for (i in files){ |
|  | print(paste("Analysing :",i), sep="") |
|  | run_fgsea(i, opt$split, as.integer(opt$rank), allLevels) |
|  | } |
|  |  |
|  | sink(format(Sys.time(), "sessionInfo-fgsea_%Y_%b_%d_%H%M%S.txt")) |
|  | sessionInfo() |
|  | sink() |
